# Topologic Reorganization of White Matter Connectivity Networks in Early-Blind Adolescents

**DOI:** 10.1155/2022/8034757

**Published:** 2022-04-28

**Authors:** Zhifeng Zhou, Long Qian, Jinping Xu, Yumin Lu, Fen Hou, Jingyi Zhou, Jinpei Luo, Gangqiang Hou, Wentao Jiang, Hengguo Li, Xia Liu

**Affiliations:** ^1^Department of Radiology, Shenzhen Mental Health Center, Shenzhen Kangning Hospital, Shenzhen 518003, China; ^2^MRI Research, GE Healthcare, Beijing 100176, China; ^3^Institute of Biomedical and Health Engineering, Shenzhen Institutes of Advanced Technology, Chinese Academy of Sciences, Shenzhen 518055, China; ^4^Department of Radiology, The First Affiliated Hospital of Guangxi University of Chinese Medicine, Nanning 530012, China; ^5^Medical Imaging Center, The First Affiliated Hospital of Jinan University, Guangzhou 510630, China

## Abstract

Blindness studies are important models for the comprehension of human brain development and reorganization, after visual deprivation early in life. To investigate the global and local topologic alterations and to identify specific reorganized neural patterns in early-blind adolescents (EBAs), we applied diffusion tensor tractography and graph theory to establish and analyze the white matter connectivity networks in 21 EBAs and 22 age- and sex-matched normal-sighted controls (NSCs). The network profiles were compared between the groups using a linear regression model, and the associations between clinical variables and network profiles were analyzed. Graph theory analysis revealed “small-world” attributes in the structural connection networks of both EBA and NSC cohorts. The EBA cohort exhibited significant lower network density and global and local efficiency, as well as significantly elevated shortest path length, compared to the NSC group. The network efficiencies were markedly reduced in the EBA cohort, with the largest alterations in the default-mode, visual, and limbic areas. Moreover, decreased regional efficiency and increased nodal path length in some visual and default-mode areas were strongly associated with the period of blindness in EBA cohort, suggesting that the function of these areas would gradually weaken in the early-blind brains. Additionally, the differences in hub distribution between the two groups were mainly within the occipital and frontal areas, suggesting that neural reorganization occurred in these brain regions after early visual deprivation during adolescence. This study revealed that the EBA brain structural network undergoes both convergent and divergent topologic reorganizations to circumvent early visual deprivation. Our research will add to the growing knowledge of underlying neural mechanisms that govern brain reorganization and development, under conditions of early visual deprivation.

## 1. Introduction

The human brain is an extremely complicated network with powerful adaptive and reorganizational capabilities. A fine example is the coordination of the complex neural plasticity processes that occur in the brain early after visual deprivation. Multiple previous studies of functional connectivity identified patterns of both increased and decreased functional connectivity in the brains of early-blind adults [1–4], especially in the visual-related areas. White matter integrity studies, using diffusion tensor imaging (DTI) method, also revealed similar structural connectivity patterns in the brains of early-blind and late-blind adults [5, 6]. This atypical pattern of brain connectivity is highly common among blind adult individuals. Despite much research, it is still unclear whether the patterns of the brain connectivity in early-blind adolescents (EBAs) and blind adults parallel one another, and how the brain develops and reorganizes after visual deprivation early in life.

Different from the regional-level approach of brain reorganization, the network-level analysis of brain connectivity provides a more comprehensive and systematic information regarding brain plasticity. In addition, the graph theory analysis method characterizes the topologic properties of brain networks, typically using both functional and structural magnetic resonance imaging (MRI) data. Among the many network analysis approaches that study brain connectivity, DTI offers a distinctive contrast that utilizes restricted directionality of water motion under anisotropic conditions. As a result, this approach is superior to others in characterizing and quantifying the white matter (WM) connectivity in the brains of blind individuals [5].

Here, we employed DTI tractography and graph theory to examine both global and regional topologic alterations and neural reorganized patterns in EBA. A recent theory, namely, reversible plasticity gradient [7], suggested that brain plasticity capacities will spontaneously decrease with age; however, it can be recovered throughout a lifespan after critical/sensitive periods. In view of this, combined with earlier work depicting impaired functional and structural connectivity in early-blind individuals, we speculated that EBAs would experience distinctly reduced topologic efficiency in WM connectivity networks, which would closely parallel clinical variables.

## 2. Materials and Methods

### 2.1. Participants

Our work received approval from the Ethics Committee of the First Affiliated Hospital of Jinan University and in compliance with the Declaration of Helsinki (1964). We have also received documented informed consent from all participants and/or guardians before magnetic resonance imaging (MRI) examinations. This study included 21 EBAs (13 males and 8 females, mean age 14.99 ± 1.97 years old, enrolled from the Guangzhou City Blind School and the clinical characteristics are listed in Table 1) with age at onset <1 year and 22 normal-sighted controls (NSCs, 11 males and 11 females, mean age 14.60 ± 2.54 years old, recruited via advertising) with matching age and sex. Given that our prior study [8] revealed a potential impact of residual light perception on the structural and functional alterations in blind brain, the EBAs eligible for this study were completely blind, with no light perception. Following are the criteria for inclusion in this study: right-handedness and between 11 and 19 years of age. Conversely, the following individuals were exempt from this study: (a) past records of head injury, psychiatric, or neurologic illnesses, (b) symptomatic or atypical neuralgia, or (c) recognizable MRI anomalies, namely, loss of myelin, vascular deformity, or tumors.

### 2.2. Image Acquisition

MRI data were obtained from the Medical Imaging Center of the First Affiliated Hospital of Jinan University. The study subjects were examined with a 3-T MR scanner (Discovery MR 750 System; General Electric, Milwaukee, WI, USA) with an 8-channel head coil. A whole-brain high-resolution T1-weighted image was obtained, using a 3-dimensional BRAVO sequence with parameters as follows: repetition time (TR) =8.2 ms, echo time (TE) =3.2 ms, flip angle =12°, field of view (FOV) =256 × 256 mm^2^, matrix size =256 × 256, slice thickness = 1 mm, and 168 slices in axial plane. DTI data was accumulated axially using a spin-echo echo-planar imaging sequence with adjusted variables as follows: TR = 6000 ms, TE = 68 ms, number of excitations = 1, FOV =256 × 256 mm^2^, matrix size =128 × 128, section thickness = 3 mm, and no intersection gap. Diffusion sensitizing gradients were applied to 75 non-collinear directions (*b* = 1000 s/mm^2^), and 5 acquisitions with no diffusion weighting (*b* = 0 s/mm^2^) were achieved. The array spatial sensitivity encoding was employed with acceleration factor of 2 to lower acquisition duration and anamorphosis. Two neuroradiologists (Z. Z and X. L with 5 and 7 years of experience, respectively) evaluated clinical abnormalities and verified image quality.

### 2.3. Data Preprocessing

The DTI information was preprocessed, according to the FSL 5.0 (https://fsl.fmrib.ox.ac.uk/fsl/fslwiki) and the Diffusion Toolkit (http://www.trackvis.org/dtk/), using the PANDA toolbox (https://www.nitrc.org/projects/panda) [9]. The process was as follows: (1) the brain mask was extracted, (2) the non-brain region within the native images was cropped, (3) head motion and eddy-current were corrected by registering the diffusion-weighted images to the b0 images with an affine transformation, and (4) the diffusion tensor was predicted via a linear least-squares fitting formula. Finally, the individual fractional anisotropy (FA) images of native space were obtained.

### 2.4. Network Construction

The brain network was created by specifying network nodes and the connections among them. Firstly, the automated anatomical labeling (AAL) template [10] enabled the parceling of the whole brain into 90 structural network nodes, with 45 nodes in each hemisphere. These regions are listed in Table 2. Next, we registered the FA images in the native space to T1-weighted images, using affine transformation. Subsequently, the structural representations were nonlinearly normalized to the ICBM152 template in standard Montreal Neurologic Institute (MNI) space. Then, the standard space was inversely warped to the native space, following the inverse warping transformation obtained from the earlier 2 steps. Finally, the AAL template in MNI space was inversely warped back to the discrete native space. At the end, all images were individually examined to ensure quality of segmentation and registration.

The amount of fiber connections between pair of nodes was described as the network edge. Whole-brain fiber tracking was conducted in individual native space with deterministic tracking algorithms. Fiber tracking termination condition was set such that the crossing angle of two consecutive moving directions was >45°or the FA was <0.2. Connectivity was detected only if the fiber number (FN) spanning two areas reached >3. This stringent threshold selection lowered the detection of pseudo-connections arising from noise or limitations in deterministic tractography [11]. Thus, a symmetric (90 × 90) FN-weighted WM connectivity network was generated for each subject.

### 2.5. Network Analysis

The topologic characteristics of the brain structural network were analyzed using graph theory [12]. Both global and nodal topological features of the brain WM connectivity networks were computed to delineate potential differences in the whole brain network between the EBA and NSC cohorts. We analyzed 8 global characteristics as listed below: network density, global efficiency (Eg), local efficiency (Eloc), clustering coefficient (Cp), shortest path length (Lp), normalized clustering coefficient (*γ*), normalized path length (*λ*), and small-world parameters (*σ*). The nodal variables included efficiency (Ne), clustering coefficient (NCp), and shortest path distance (NLp). A description of each parameter is summarized in supplementary materials Table S1. Additionally, hub nodes were described as the 13 (15%) top-ranking nodes with the largest mean nodal degrees among all brain regions within each cohort [13]. The GRETNA toolbox (https://www.nitrc.org/projects/gretna) [14] was employed for all network analyses and the BrainNet Viewer software (https://www.nitrc.org/projects/bnv/) [15] was employed for visualization.

### 2.6. Statistical Analysis

Statistical analyses were done with the Statistical Package for Social Science (SPSS Version 23.0; https://www.spss.com/). Two-sample t- and chi-square tests were used for differences between age and sex, respectively. Linear regression analyses were completed to delineate differences in topologic networks, with age and sex as covariates. Multiple comparisons were conducted with Bonferroni correction at *p* < 0.05. For network parameters harboring marked cohort differences, partial correlation analyses were completed between the parameters and blind duration, after adjusting for age and sex in the EBA cohort.

## 3. Results

Thirteen men and 8 women were included in the EBA cohort, while the NSC cohort had 11 men and 11 women. No discernible changes were observed in age and sex between the two cohorts (*p* > 0.05). Table 1 summarizes the clinical information of all EBAs and Table 3 reports the demographics of both EBA and NSC cohorts.

### 3.1. Global Differences in WM Structural Networks between Groups

**Figure 1:**
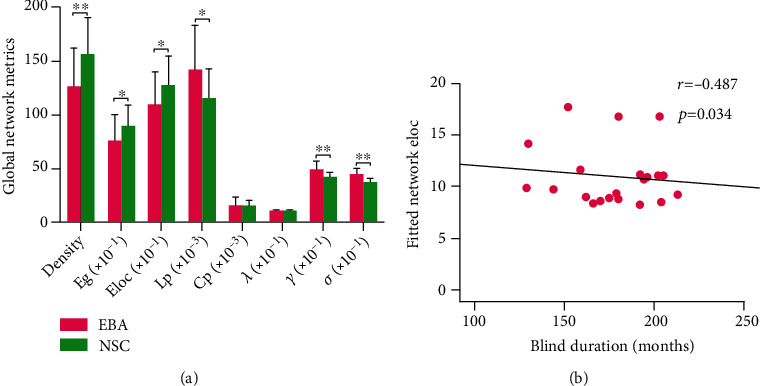
Group differences in global network metrics of WM structural networks (a) and the correlation between network Eloc and blind duration in the EBA cohort (b). (a) Bar charts and error bars represent the mean values and standard deviations, respectively (EBA: *n* = 21; NSC: *n* = 22). ∗*p* < 0.05 (Bonferroni corrected); ∗∗*p* < 0.001 (Bonferroni corrected). (b) Scatterplots show the significantly negative correlation between network Eloc and blind duration in the EBA cohort (*n* = 21). The fitted values indicate the residuals of original values of Eloc adjusted age and sex and corrected with mean value. Abbreviations: Eg: global efficiency; Eloc: local efficiency; Lp: shortest path length; Cp: clustering coefficient; *λ*: normalized path length; *γ*: normalized clustering coefficient; *σ*: small-world parameters; EBA: early-blind adolescents; NSC: normal-sighted controls.

The EBA cohort exhibited markedly low network density (*t* = -3.78, *p* < 0.001, corrected), Eg (*t* = -2.87, *p* = 0.007, corrected), and Eloc (*t* = -2.67, *p* = 0.011, corrected), relative to the NSC cohort (Figure 1(a)).

### 3.2. Small-Worldness Differences in the WM Structural Networks between Groups

Using graph-theoretical analyses, we discovered that both cohorts displayed high normalized clustering coefficients and similar normalized path length (*γ* > 1, *λ* ≈ 1, and *σ* > 1) in their WM structural networks, indicating the presence of distinctive small-world organizations in both cohorts. Moreover, EBA cohorts exhibited longer Lp (*t* = 3.41, *p* = 0.002, corrected), higher normalized clustering coefficient (*t* = 4.56, *p* < 0.001, corrected), and higher small-world parameters (*t* = 5.74, *p* < 0.001, corrected) than the NSC cohort (Figure 1(a)). There were no marked alterations between the two cohorts in terms of clustering coefficient (*t* = 0.35, *p* = 0.73) and normalized path length (*t* = -1.10, *p* = 0.28).

### 3.3. Intercohort Differences in Regional Efficiency

**Figure 2:**
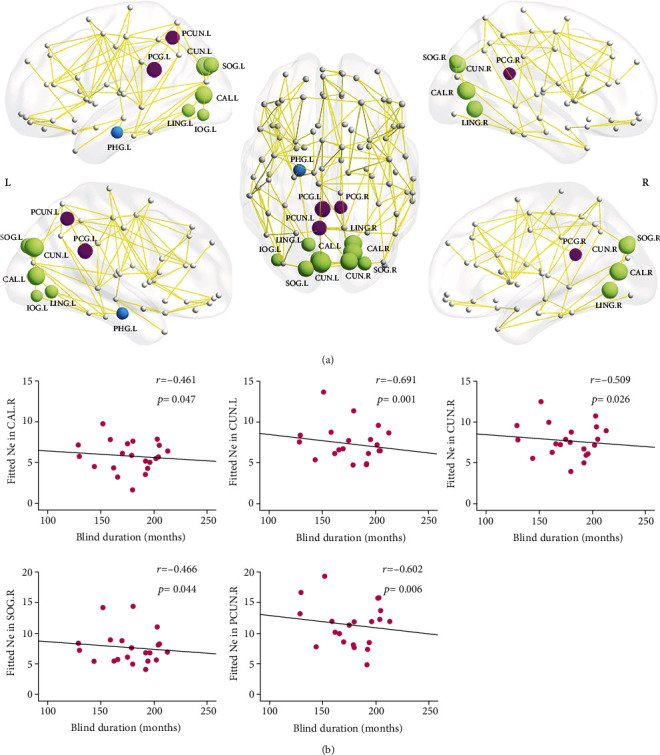
Distribution of brain regions with significant intercohort differences in nodal efficiency (Ne) (a) and the correlation with blind duration in the EBA cohort (b). (a) Regions with decreased Ne are represented in different colors: purple for the default-mode system, green for the visual system, and blue for the limbic system. The node sizes indicate the significance of intercohort differences in Ne. (b) Scatterplots show the significantly negative correlation between Ne and blind duration in EBA cohort (*n* = 21). The fitted values indicate the residuals of original values of Ne adjusted age and sex and corrected with mean value. The abbreviations of nodes can be referred in Table 4.

**Figure 3:**
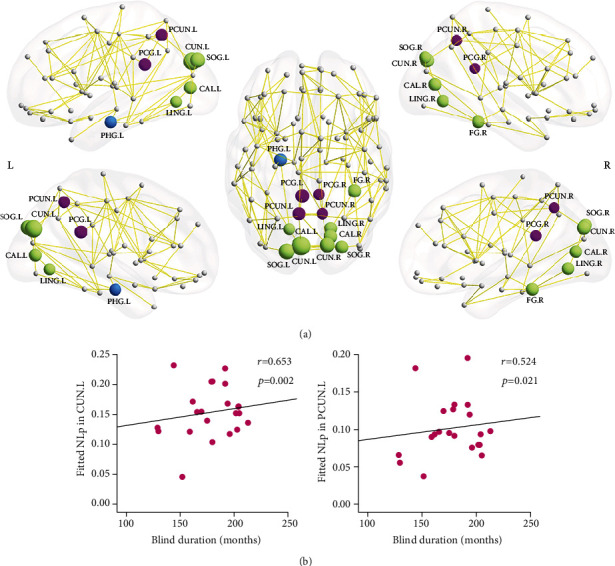
Distribution of brain regions with significantly intercohort differences in nodal shortest path length (NLp) (a) and the correlation with blind duration in EBA cohort (b). (a) Regions with longer NLp are represented in different colors: purple for the default-mode system, green for the visual system, and blue for the limbic system. The node sizes indicate the significance of intercohort difference in NLp. (b) Scatterplots show a significantly positive correlation between NLp and blind duration in EBA cohort (*n* = 21). The fitted values indicate the residuals of original values of NLp adjusted age and sex and corrected with mean value. The abbreviations of nodes can be referred in Table 4.

The EBA cohort had lower Ne in the bilateral posterior cingulate gyri, calcarine cortices, cuneus, lingual gyri, superior occipital gyri, left precuneus, left parahippocampal gyrus, and left inferior occipital gyrus (*p* < 0.05, corrected) (Table 4, Figure 2(a)). Apart from the left inferior occipital gyrus, most regions, including the right fusiform gyrus and right precuneus, showed significantly longer Nlp (*p* < 0.05, corrected) in the EBA cohort, relative to the NSC cohort (Table 4, Figure 3(a)). Based on the functionality of these brain areas, they were classified into 3 categories: (1) the default-mode system that included bilateral posterior cingulate gyri and precuneus; (2) the visual system that included bilateral calcarine cortices, cuneus, lingual gyri, superior occipital gyri, left inferior occipital gyrus, and right fusiform gyrus; and (3) the limbic system that included the left parahippocampus. Additionally, some brain regions showed moderately significant NCp alterations in the EBA cohort, relative to NSCs, particularly, in the bilateral middle frontal gyri and left triangular parts of inferior frontal gyrus and decreased NCp in right supplementary motor area, right parahippocampus, and bilateral cuneus (*p* < 0.05, uncorrected; Supplementary Materials Table S2, Figure S1).

### 3.4. Hub Regions

**Figure 4:**
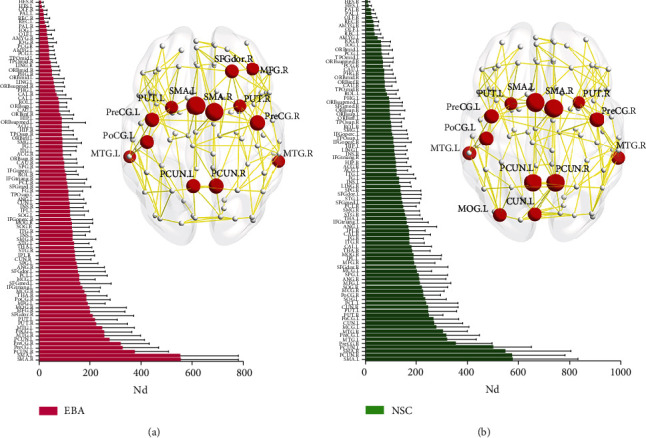
Hub region distributions (top 13) in the WM structural network in EBA (a) and NSC (b) cohorts. The left panels indicate the 90 brain regions of AAL atlas sorted by mean nodal degree (Nd) in ascending order for each cohort. The right panels show hub distributions of each cohort. The hub nodes are shown in red, and node sizes indicate the significance of intercohort difference in Nd. The abbreviations of brain regions are presented in Table 2.

Based on the networks of each cohort, similar hub distribution patterns were found in the EBA and NSC cohorts. These highly similar hub locations included the bilateral precentral gyri, supplementary motor areas, precuneus, middle temporal gyri, putamen, and left postcentral gyri (Figure 4). However, two regions, i.e., the left cuneus and middle occipital gyrus, were recognized as hubs in the NSC cohort alone, and hubs in the right dorsolateral part of superior frontal gyrus and right middle frontal gyrus were unique to the EBA cohort alone.

### 3.5. Correlation between Network Efficiency and Blind Duration

In the EBA cohort, the network Eloc was inversely associated with the period of blindness (*r* = -0.487, *p* = 0.034) (Figure 1(b)). Similarly, the Ne of some regions, including the right calcarine cortex (*r* = -0.461, *p* = 0.047), bilateral cuneus (left: *r* = -0.691, *p* = 0.001; right: *r* = -0.509, *p* = 0.026), right superior occipital gyrus (*r* = -0.466, *p* = 0.044), and left precuneus (*r* = -0.602, *p* = 0.006), were also inversely related to the period of blindness. The Nlp of the left cuneus (*r* = 0.653, *p* = 0.002) and left precuneus (*r* = 0.524, *p* = 0.021) was positively associated with the period of blindness (Figures 2(b) and 3(b)).

## 4. Discussion

Using DTI tractography and graph theory, we explored the WM structural networks of the EBAs and NSCs brains. Structural connection matrices examined in these cohorts displayed “small-world” characteristics. The EBA cohort exhibited significantly lower network density, Eg, and Eloc, as well as significantly increased Lp, compared to the NSC cohort. In detail, the regional efficiencies of the default-mode, visual, and limbic areas were markedly reduced in the EBA cohort, relative to the NSC cohort. Moreover, decreases in Ne and increases in NLp within certain visual and default-mode regions were strongly associated with the period of blindness in the EBA cohort, indicative of a gradual decline in the function of these areas in the early-blind brain during adolescence. Additionally, the differences in hub distribution between the two groups were mainly localized within the occipital and frontal areas, suggesting that neural reorganization occurred in these brain regions after early visual deprivation during the adolescence. Taken together, our findings indicate topological reorganization of the WM connectivity networks in EBAs, relative to NSCs. Additionally, these alterations can gradually accumulate over prolonged duration of blindness.

### 4.1. Small-World Efficiencies in the EBA WM Network

The human brain is a complicated network of balanced global integration and local segregation processes. Here, we established the small-world features of EBA and NSC structural networks, characterized by a rapid, efficient, low-cost information flow through a complex network [16]. The conclusions of this study are in accordance with other research on blind children [17] and adults [18].

Although small-world properties were detected in both EBA and NSC networks, the Eg and Eloc were markedly reduced in the EBA networks, relative to the NSCs. The Eg, calculated from the average inverse Lp between remote brain locations, represents the ability of long-range information transmission. In contrast, the Eloc reflects the ability of short-range information transmission, which is mainly associated with the connections between neighboring brain regions. Conversely, reduced Eg and Eloc represent abnormal topological networks reorganization in EBA, likely caused by the delayed or defective development of structural connections due to the absence of visual stimuli. Our findings are also supported by multiple other DTI studies, which demonstrated dysregulated micro-structural integrity in the posterior visual radiation, occipital and temporal thalamocortical projections, and visual processing stream in early-blind individuals [6, 8, 19, 20].

### 4.2. Altered Regional Efficiency in the WM Network of EBA

We demonstrated lower Ne and higher NLp within EBA networks. The affected locations were classified into three discrete systems: default-mode, visual, and limbic systems.

#### 4.2.1. Default-Mode System

EBA networks had reduced regional efficiency in the core components of the default-mode network, namely, the bilateral posterior cingulate gyri and precuneus. These locations primarily depend on visual input and are involved in attentive behavior [21, 22] and in integrating visuospatial information in sighted controls [23–25]. In EBAs, however, the function of these areas may be diminished by the lack of necessary visual input, which may lead to the reduced Ne in the corresponding regions. Likewise, using high angular resolution diffusion imaging, Bauer et al. [3] indicated decreased structural connectivity between posterior cingulate and frontal cortices and between precuneus and parietal cortices, in early-onset blind adults. In other words, the alterations in the structural connectivity of the blind brain's default-mode system appear well before adulthood. Additionally, in our study, we discovered that Ne and NLp of the left precuneus were negatively and positively associated with the period of blindness in EBA, respectively, suggesting that the function of the left precuneus gradually weaken over time in the early-blind brain during adolescence.

#### 4.2.2. Visual System

Low regional efficiencies were also observed in most occipital areas in EBA, including the primary visual regions (calcarine, aka the striate cortex), which predominantly process the visual input from the retina, and the visual association areas (the cuneus, lingual, fusiform, superior occipital gyrus, and inferior occipital gyrus; all parts of the extrastriate visual cortex), and are mainly involved in the subsequent steps of visual input processing. These findings are supported by the disuse atrophy hypothesis of the visual cortex in early-blind brains [26]. Similarly, anatomical studies have also revealed widespread abnormal anatomical alterations, such as thin cortical thickness and reduced cortical and WM volume, in the occipital cortices of the early blind [3, 19, 27–29]. Correspondingly, reduced functional connections were reported between the visual areas and non-visual sensory [1, 3, 30–32], somatosensory [1–3, 31, 33], or motor cortices [1, 2, 31], as well as between primary visual areas and visual association areas [30, 34], in early-blind brains. Nevertheless, our results showed a broader range of alterations in the structural connections of the visual system than previously found in adults with early blindness. This may be the result of some connectivity pathways in the occipital lobe that are not dependent on visual experience developed during adulthood with increased experiential input [35, 36], thus masking some structural and functional impairments in the occipital lobe, due to lack of visual input. In addition, we found that the Ne of the right calcarine, bilateral cuneus, and right superior occipital gyrus were all inversely associated with the period of blindness in EBA. These data indicate a structurally progressive and cumulative defect in the visual system of early blind during adolescence.

#### 4.2.3. Limbic System

The parahippocampal gyrus is an important region within the limbic system and is involved in multiple high-level cognitive processes [37, 38]. Moreover, the bilateral parahippocampal gyrus showed asymmetric functional preferences. In particular, the left parahippocampus was more likely to process semantic and language information [39–41], while the right parahippocampus was often activated during spatial navigation tasks processing [42–44]. This functional asymmetry of the bilateral parahippocampal gyrus was particularly pronounced in blind brains. For instance, the neural activity patterns associated with semantic processing in the left parahippocampus in blind adults were highly similar to those in the sighted controls, even though they were activated via verbal and auditory stimuli in blind subjects while activated by visual, verbal, and auditory stimuli in sighted individuals [45]. However, in a tactile spatial navigation task, the responses of the right parahippocampus were significantly increased in congenital blind subjects, but not in blindfolded sighted participants [46], which suggests that the right parahippocampus of blind individuals performs better at integrating visual independent multimodal information than that of sighted controls. From the perspective of neurodevelopment, a recent myelin water imaging study [47] reported increasing myelination from early adolescence to adulthood in the right parahippocampal region, but not in the left parahippocampus. In the present study, we demonstrated significantly reduced regional efficiency in the left parahippocampal gyrus of EBAs versus NSCs. Determining whether this is the result of natural neural development or neural reorganization after visual deprivation, or a combination of the previous two reasons, would require further investigation.

### 4.3. Reorganization of Hubs in the EBA WM Network

The nodal degree measurements enable us to recognize the most essential nodes (i.e., with the most connections to other nodes) within the brain network. Here, we discovered similar hub distribution patterns in both cohorts, which is in accordance with another study [18]. Most hub regions, such as the bilateral precentral gyri, supplementary motor areas, precuneus, putamen, middle temporal gyri, and left postcentral gyrus, were the same in both EBA and NSC cohorts, and belonged to the association cortex, which receives signals from various other cortical locations [48]. Similar to our conclusion, several prior studies have also identified the association cortex as a crucial location for the structural and functional brain networks in humans [49–51]. But, relative to the NSC cohort, the EBA cohort was missing two hub locations, namely, the left cuneus and left middle occipital gyrus, indicating low usage of these locations in the EBA brain. Interestingly, two additional hub regions, namely, the right dorsolateral part of superior frontal gyrus and right middle frontal gyrus, were found to be of significance in the EBA cohort, but not the NSC cohort, indicating increased usage of these locations in the EBA brain. In view of this, we speculate that early visual deprivation may promote neural plasticity in the frontal and occipital related areas of the early blind brain.

### 4.4. Limitations

Our work exclusively examined WM structural networks. In future investigations, a combined examination of structural and functional connectivity networks would facilitate a thorough comprehension of the neural reorganizations in the EBA. Moreover, the DTI-based tractography method may be affected by the crossing fibers. Thus, a more accurate model is warranted for the calculation of WM connections to verify the results of this study in the future.

## 5. Conclusion

In conclusion, our study revealed both convergent and divergent topologic reorganizations within the EBA brain structural network. From a network perspective, our work sheds light on the patterns of the brain development and reorganization during adolescence in blind individuals that experiences early visual deprivation. The present findings will provide further insight into the study of neural reorganization and development under visual deprivation.

## Figures and Tables

**Table 1:** Clinical characteristics of blind participants.

Subjects	Sex	Age (years)	Onset	Blind duration (months)	Causes of blindness
EBA01	M	14	Age of 3rd month	170	ROP
EBA02	M	16	Age of 2nd month	194	ROP
EBA03	M	11	Age of 2nd month	130	ROP
EBA04	F	14	Age of 1st month	166	ROP
EBA05	M	17	At birth	202	Unknown
EBA06	M	15	At birth	180	Unknown
EBA07	F	12	Age of 3rd month	144	ROP
EBA08	F	16	Age of 3rd month	192	ROP
EBA09	M	17	At birth	203	CG
EBA10	F	17	At birth	205	ROP
EBA11	M	15	At birth	180	ROP
EBA12	M	18	At birth	213	ROP
EBA13	M	16	At birth	192	Unknown
EBA14	M	17	At birth	204	Unknown
EBA15	F	15	Age of 2nd month	152	ROP
EBA16	F	17	Age of 1st month	196	ROP
EBA17	M	15	Age of 4th month	175	ROP
EBA18	M	15	At birth	179	Unknown
EBA19	F	11	Age of 3rd month	129	ETE
EBA20	M	13	At birth	159	Unknown
EBA21	F	14	Age of 3rd month	162	ROP

Abbreviations: ROP: retinopathy of prematurity; CG: congenital glaucoma; ETE: eyeball excision for tumor; EBA: early-blind adolescents; M: male; F: female.

**Table 2:** Cortical and subcortical regions of interest defined in this study.

Regions	Abbreviations	Regions	Abbreviations
Precentral gyrus	PreCG	Lingual gyrus	LING
Superior frontal gyrus, dorsolateral	SFGdor	Superior occipital gyrus	SOG
Superior frontal gyrus, orbital part	ORBsup	Middle occipital gyrus	MOG
Middle frontal gyrus	MFG	Inferior occipital gyrus	IOG
Middle frontal gyrus, orbital part	ORBmid	Fusiform gyrus	FG
Inferior frontal gyrus, opercular part	IFGoperc	Postcentral gyrus	PoCG
Inferior frontal gyrus, triangular part	IFGtriang	Superior parietal gyrus	SPG
Inferior frontal gyrus, orbital part	ORBinf	Angular gyri	IPL
Rolandic operculum	ROL	Supramarginal gyrus	SMG
Supplementary motor area	SMA	Angular gyrus	ANG
Olfactory cortex	OLF	Precuneus	PCUN
Superior frontal gyrus, medial	SFGmed	Paracentral lobule	PCL
Superior frontal gyrus, medial orbital	ORBsupmed	Caudate nucleus	CAU
Rectus gyrus	REC	Lenticular nucleus, putamen	PUT
Insula	INS	Lenticular nucleus, pallidum	PAL
Anterior cingulate and paracingulate gyri	ACG	Thalamus	THA
Median cingulate and paracingulate gyri	MCG	Heschl gyrus	HES
Posterior cingulate gyrus	PCG	Superior temporal gyrus	STG
Hippocampus	HIP	Temporal pole: Superior temporal gyrus	TPOsup
Parahippocampal gyrus	PHG	Middle temporal gyrus	MTG
Amygdala	AMYG	Temporal pole: Middle temporal gyrus	TPOmid
Calcarine fissure and surrounding cortex	CAL	Inferior temporal gyrus	ITG
Cuneus	CUN		

**Table 3:** Demographics and clinical characteristics of all participants.

Characteristics	EBA	NSC	Statistics	*p* values
Age (mean ± sd) (years)	14.99 ± 1.97	14.60 ± 2.54	0.565	0.570
Sex (M/F)	13/8	11/11	0.617	0.432
Blind duration (mean ± sd) (months)	177.48 ± 24.68	NA	NA	NA

*Note*: Age was reported by participants or guardians at the time of MR imaging. Abbreviations: EBA: early-blind adolescents; NSC: normal-sighted controls; NA: not applicable.

**Table 4:** Brain regions with significant nodal differences between groups.

Systems	Regions	Abbreviations	Ne		NLp	
			*T* values	*p* values	*T* values	*p* values
Default-mode	Left posterior cingulate gyrus	PCG.L	-5.10	<0.001	4.71	<0.001
	Right posterior cingulate gyrus	PCG.R	-4.04	<0.001	3.81	<0.001
	Left precuneus	PCUN.L	-4.61	<0.001	4.19	<0.001
	Right precuneus	PCUN.R	NA	NA	3.76	0.001
Visual	Left calcarine	CAL.L	-6.24	<0.001	4.81	<0.001
	Right calcarine	CAL.R	-6.43	<0.001	4.60	<0.001
	Left cuneus	CUN.L	-6.87	<0.001	6.72	<0.001
	Right cuneus	CUN.R	-5.95	<0.001	5.33	<0.001
	Left lingual	LING.L	-4.38	<0.001	4.02	<0.001
	Right lingual	LING.R	-5.81	<0.001	4.32	<0.001
	Left superior occipital gyrus	SOG.L	-5.26	<0.001	5.67	<0.001
	Right superior occipital gyrus	SOG.R	-4.40	<0.001	4.31	<0.001
	Left inferior occipital gyrus	IOG.L	-3.84	<0.001	NA	NA
	Right fusiform gyrus	FG.R	NA	NA	4.45	<0.001
Limbic	Left parahippocampal gyrus	PHG.L	-3.82	<0.001	4.20	<0.001

*Note*: The regions with significant group differences in nodal efficiency (Ne) and nodal shortest path (NLp) at *p* < 0.05 (Bonferroni-corrected) can be categorized into 3 functional systems. Abbreviations: EBA: early-blind adolescents; NSC: normal-sighted controls; NA: not applicable.

## Data Availability

The data that support the findings of this study are available on request from the corresponding author Dr. Xia Liu (auraliu0061@126.com). The data are not publicly available due to privacy or ethical restrictions.

## Supplementary Materials

Supplementary Materials Supplemental Table S1: Definitions and interpretations of global and nodal network metrics. Supplemental Table S2: Brain regions with moderate differences of NCp between EBA and NSC groups. Supplemental Figure S1: Brain regions with moderate differences in NCp in EBA cohort.
