# Supplementary material for: Topologic Reorganization of White Matter Connectivity Networks in Early-Blind Adolescents
Source: Neural Plast. 2022 Apr 28;2022:8034757. doi: 10.1155/2022/8034757 (PMC9072039; doi:10.1155/2022/8034757)
Supplement: Supplementary Materials — Supplemental Table S1: Definitions and interpretations of global and nodal network metrics. Supplemental Table S2: Brain regions with moderate differences of NCp between EBA and NSC groups. Supplemental Figure S1: Brain regions with moderate differences in NCp in EBA cohort. [file 8034757.f1.docx]

**Topologic Reorganization of White Matter Connectivity Networks in Early-Blind Adolescents**

**Supplemental Materials**

Supplemental Table

**Table S1** Definitions and interpretations of global and nodal network metrics

| Metrics | Definitions | Notes |
| --- | --- | --- |
| Network density | $\frac{1}{N}\sum_{j=1}^{N} \#Ej$ | *N* is the total number of nodes in the network, *#Ej* is the number of edges connecting the neighbors of node *j*. |
| Global efficiency | $Eg=\frac{1}{N(N-1)}\sum_{i\neq j} \frac{1}{min\{L_{i,j}\}}$ | *Eg* values the efficiency of information integration through the whole network |
| Local efficiency | $Eloc=\frac{1}{N}\sum_{i\neq j} Eg(Gi)$ | *Gi* denotes the subgraph composed of the direct-linked neighbors of node *i*. *Eloc* measures the ability of fault tolerance of the network. |
| Clustering coefficient | $Cp=\frac{1}{N}\sum_{j=1}^{N} \frac{{\#E}_{j}}{\#V_{j}(\#V_{j}-1)/2}$ | *#Vj* is the number of neighbors of node *j*. *Cp* reflects the extent of local clustered connectivity of a network. |
| Shortest path length | $Lp=\frac{1}{N(N-1)}\sum_{i\neq j} min\{L_{i,j}\}$ | *Min{L_i,j_}* is the minimal number of edges between node *i* and node *j*. *Lp* reflects the ability of information propagation in the network. |
| Normalized clustering coefficient | γ =${C_{p}^{real}}/{C_{p}^{rand}}$ | $C_{p}^{real} and C_{p}^{rand}$are the average *Cp* of the real given network and 100 random networks, respectively, with the same number of nodes, number of edges and degree distribution as the real network. |
| Normalized path length | λ = ${L_{p}^{real}}/{L_{p}^{rand}}$ | $L_{p}^{real} and L_{p}^{rand}$ are the average *Lp* of the real given network and 100 random networks, respectively, with the same number of nodes, number of edges and degree distribution as the real network. |
| Small world parameters | *σ* = *γ* / *λ* | *σ* indicates how random or ordered a network is. The *σ* is typically > 1 for small-world networks. |
| Nodal efficiency | $Ne_{i}=\frac{1}{(N-1)}\sum_{i\neq j} \frac{1}{min\{L_{i,j}\}}$ | Nodal efficiency of node *i* measures the information propagation efficiency from node *i* to other nodes in the network |
| Nodal clustering coefficient | $NCp_{j}=\sum_{j=1}^{N} \frac{{\#E}_{j}}{\#V_{j}(\#V_{j}-1)/2}$ | The clustering coefficient of node *j* is a ratio that the possible connections to the actually exist connections between the nearest neighbors of node *j*. *NCp* reflect the clustered connectivity around a given node in the network |
| Nodal shortest path length | $NLp_{i,j}=\frac{1}{(N-1)}\sum_{i\neq j} min\{L_{i,j}\}$ | *NLp_i,j_* is the minimal number of edges between node *i* and node *j*. It is inversely proportional to the ability of transmit information of a node in the network. |

| **Table S2** Brain regions with moderate differences of NCp between EBA and NSC groups | | | | | | | |
| --- | --- | --- | --- | --- | --- | --- | --- |
| Regions | Abbreviations | EBA | | NSC | | T values | p values (uncorrected) |
|  |  | mean | sd | mean | sd |  |  |
| Left middle frontal gyrus | MFG.L | 0.046 | 0.031 | 0.025 | 0.022 | 2.470 | 0.018 |
| Right middle frontal gyrus | MFG.R | 0.029 | 0.028 | 0.017 | 0.008 | 2.174 | 0.036 |
| Left inferior frontal gyrus, triangular part | IFGtriang.L | 0.025 | 0.015 | 0.015 | 0.012 | 2.591 | 0.013 |
| Right supplementary motor area | SMA.R | 0.022 | 0.007 | 0.031 | 0.016 | -2.210 | 0.033 |
| Right parahippocampal gyrus | PHG.R | 0.009 | 0.007 | 0.016 | 0.009 | -2.617 | 0.013 |
| Left cuneus | CUN.L | 0.022 | 0.018 | 0.034 | 0.014 | -2.289 | 0.028 |
| Right cuneus | CUN.R | 0.022 | 0.012 | 0.032 | 0.012 | -2.543 | 0.015 |
| Abbreviations: EBA; early-blind adolescents; NSC: normal-sighted controls. | | | | | | | |
|  | | | | | | | |


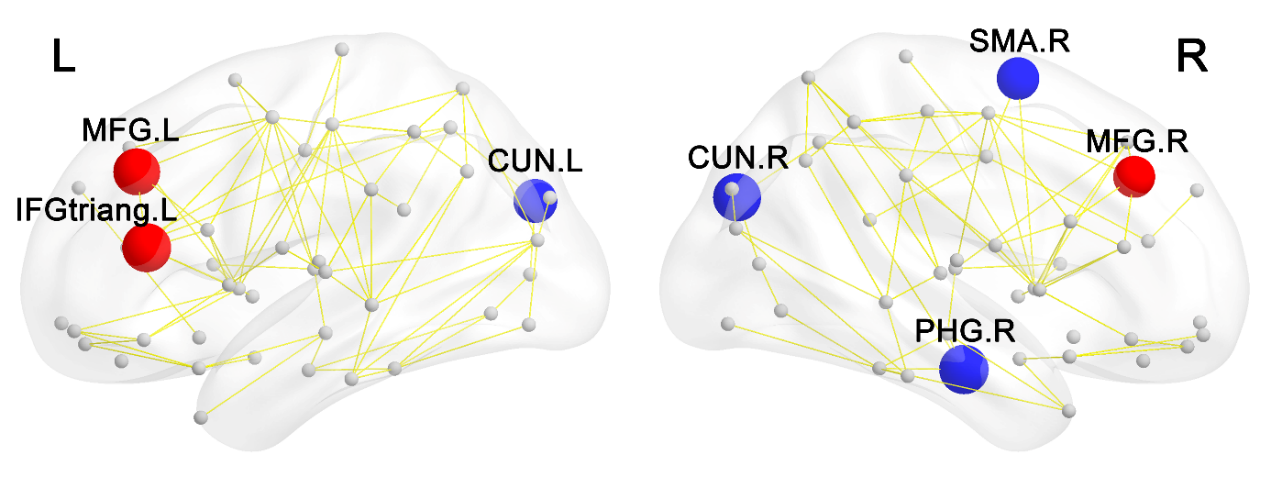
 **Figure S1:** Brain regions with moderate differences in nodal clustering coefficient (NCp) in EBA cohort (p < 0.05, uncorrected). The regions with significantly enhanced NCp are colored in red, and those with decreased NCp are colored in blue. The node sizes indicate the significance of intercohort differences in NCp. The abbreviations of nodes can be referred in Table S2.
